# *erbB2* Overexpression in Uterine Serous Cancer: A Molecular Target for Trastuzumab Therapy

**DOI:** 10.1155/2011/128295

**Published:** 2011-08-11

**Authors:** Karim S. ElSahwi, Alessandro D. Santin

**Affiliations:** Department of Obstetrics, Gynecology & Reproductive Sciences, Yale University School of Medicine, P.O. Box 208063, New Haven, CT 06520-8063, USA

## Abstract

Endometrial cancer is the most common female genital tract malignancy in the United States. Type I endometrial cancer is usually diagnosed at an early stage, and has a good prognosis. Type II is very aggressive, and is responsible for most uterine cancer relapses and deaths. Uterine serous adenocarcinomas (USC) constitute the majority of Type II variants. They have a higher propensity for lymph node and distant metastases. They are frequently aneuploid and associated with p53 mutations. erbB2 overexpression in USC has been described. The incidence, which is higher in African Americans, ranges from 18–80%. erbB2 overexpression was found to be associated with higher stage, chemoresistance, and worse survival. Trastuzumab a humanized mAb was approved by the FDA for treatment of breast cancers that overexpress erbB2 in combination with standard chemotherapy. Evidence of trastuzumab activity in USC has been reported in vitro, as well as in case reports of advanced and recurrent cases. Promising results were obtained in these heavily pretreated patients either with trastuzumab alone or in combination with chemotherapy. This supports the hypothesis that trastuzumab may very well be an attractive and viable treatment option for advanced stage USC tumors that overexpress the erbB2, and is worthy of further study.

## 1. Endometrial Cancer

Endometrial cancer is the most common female genital tract malignancy in the United States, with an incidence of 40100 new cases and 7470 deaths annually [1]. Type I endometrial cancer, which has an endometrioid histology, is associated with obesity, estrogen excess, and is heralded by endometrial hyperplasia. It is usually diagnosed at an early stage and has a good prognosis. On the other hand, Type II endometrial cancer is a very aggressive variant of the disease and is responsible for about 50% of all uterine cancer relapses and most deaths [2].

## 2. Uterine Serous Cancer

Uterine serous adenocarcinomas (USC) constitute the majority of Type II variants, and about 10% of all endometrial cancers [3]. USC has a higher propensity for lymphovascular invasion, and intraperitoneal as well as extraabdominal spread, than endometrioid carcinoma. It also has a significantly greater incidence of pelvic and para-aortic lymph node metastases [4]. At the time of presentation, approximately 60 to 70 percent of women with USC will have-disease spread outside of the uterus [3]. Overall 1-year-, 2-year-, and 5-year-survival in USC is 84%, 71%, and 54%, respectively. This is compared to a respective overall survival of 94%, 89%, and 80% in endometrioid adenocarcinoma [5]. In a Yale series, there was no survival difference between stage I patients who had 10–50% of the endometrial carcinoma composed of USC compared with those with more than 50% USC [6].

Surgical staging remains the mainstay of treatment of USC, as the majority of patients with disease clinically confined to the uterus will be upstaged (57–70%) Staging entails a hysterectomy, bilateral salpingo-oopherectomy, omentectomy, bilateral pelvic lymphadenectomy, para-aortic node sampling, and peritoneal cytology obtained upon entry into the abdominal cavity. Adjuvant therapy is usually recommended [7]. 

A Gynecologic Oncology Group trial (GOG 94) reported a 35% 5-year-disease-free survival when 31 women with stages I and II USC received adjuvant postoperative whole abdomen radiation therapy. Some institutional experiences suggest that whole abdomen radiation therapy may be beneficial. Others have not found it to be effective [6, 8, 9]. Martin et al. hypothesized that USC radiation resistance may be attributed to *p53* overexpression through the evasion of radiation-induced apoptosis. Indeed, radioresistance of breast cancer has been associated with *erbB2* overexpression [9]. The Yale experience suggests that vaginal apex brachytherapy plays an important part in the management of the disease, as no recurrences presented at the vaginal apex among 43 stage I patients with early stage USC treated with this technique [6].

Platinum-based chemotherapy has been routinely employed in the management of both early and advanced stages of USC. Although conflicting data exist, a Yale series suggested that carboplatin and paclitaxel in surgical stage I USC should be employed as part of the routine method of management of the disease [6]. Only one of 29 stage IA–IC patients (3.4%) recurred, who received platinum-based chemotherapy, whereas 20 out of 32 (62.5%) who did not receive chemotherapy recurred [6]. Recently, a study of 25 stage I-II USC patients treated at Sloan-Kettering memorial hospital with acombination of carboplatin/paclitaxel and vaginal brachytherapy found comparable results [10]. The overall survival of women with USC, however, remains about 30%. The survival of women with stages I-II USC is 35–50% and for stages III-IV is 0–15% [11]. Overall recurrence rate is about 80%, and new therapeutic modalities are still needed [12].

## 3. *erbB2* in USC

Whereas USC does not share the typical risk factors known to influence its endometrioid counterpart, several unique prognosticators have been identified in this variant. Commonly seen in older, thin, and nulliparous patients, and in African American women, the molecular biology of USC is also distinctive. The tumors, infrequently express estrogen (ER) and progesterone (PR) receptors, are frequently aneuploid, and the majority (90%) are associated with *p53* mutations [13]. *P53* mutation is known to enhance the aggressiveness of the disease by modulating pathways of proliferation and apoptosis. 

In 2002, Santin et al. reported for the first time on *erbB2* overexpression in USC specimens in vitro. Eight out of 10 specimens stained heavily (2+, 3+) for *erbB2* by immunohistochemistry. Furthermore, USC cell lines expressed 10-fold higher *erbB2* levels than *erbB2*-positive ovarian and breast cancer cell lines by flowcytometry. The sensitivity of those cell lines to antibody dependent cytotoxicity in vitro was also demonstrated in this study [14].

The same group then used oligonucleotide microarrays to analyze gene expression profiling of USC and normal endometrial cells in culture. Among 529 differentially expressed genes, *c-erbB2* gene which, encodes for the *erbB2* receptor, was found to be highly expressed in USC [15]. 

In later publications, the same group, as well as others, have shown that *erbB2* receptor is expressed in 18–62% of USC. USC overexpression and amplification was found to be associated with chemoresistance, higher stage, and worse overall survival [12, 14, 16, 17]. Indeed, *erbB2* gene amplification by FISH was observed more frequently in African American patients compared to Caucasians (*P* = 0.02), and to be associated with a worse prognosis in this subgroup of patients (*P* = 0.01). It was associated with a shorter overall survival in all patients tested (*P* = 0.0008) [18].

The correlation between *erbB2* overexpression by immunohistochemistry (IHC) and *erbB2* gene amplification by fluorescent in situ hybridization (FISH) was addressed in two further studies. In one study, moderate to strong expression of *erbB2* was found in 16 (62%) of 26 USC samples evaluated, with 7 (27%) samples showing moderate staining (2+) and 9 (35%) showing strong staining (3+). Amplification of the *erbB2* gene by FISH was observed in 11 (42%) of the 26 samples. Protein overexpression and gene amplification were found to correlate in 100% (9 of 9) of the 3+ tumors and in 29% (2 of 7) of the 2+ tumors. None of the 10 USC samples that scored 0 or 1+ by IHC tested positive for gene amplification by FISH [19]. In another study, strong *erbB2* membrane staining (3+) of USC samples was observed in 16.6% (2 of 12) of samples tested. There was perfect correlation with gene amplification by FISH, as well as messenger RNA expression by quantitative real time reverse transcription (RT) polymerase chain reaction (PCR) [16]. Interpretation of IHC results follow the breast cancer guidelines, where a 3+ score is defined as heavy staining of the cell membrane in more than 10% of tumor cells, 2+ is defined as moderate staining in more than 10% of cells, and a score of 1+ or 0 is defined as light or no membrane staining, respectively. A score of 1+ or 0 indicates a negative test. We have also adopted the same treatment strategy used in breast cancer, where confirmation of an IHC 2+ score by FISH testing, performed before treatment with trastuzumab, is begun.

A Gynecologic Oncology Group (GOG) analysis of 234 samples of advanced and recurrent endometrial cancer found *erbB2* overexpression and gene amplification in 44% and 12% of cases, respectively. There was a significant increased frequency of overexpression in USC versus all others (61% versus 41%) [20]. Neither protein overexpression nor gene amplification predicted overall survival, however, the study included highly advanced cases, and the analysis combined endometrioid and serous cancers (i.e., Type I and Type II endometrial carcinomas).

## 4. *erbB2*

*erbB2* is a member of the erbB receptor tyrosine kinase. This is a family of 4 transmembrane glycoproteins (EGFR/erbB1, *erbB2*, erbB3, and erbB4) that are expressed on epithelial, mesenchymal, and neuronal cells. erbB receptors are activated in response to binding with 11 ligands produced in an autocrine fashion in the individual cells or a paracrine fashion in the surrounding tissue. Ligand binding results in dimerization of the receptor either with a twin receptor (homodimerization) or with one of its siblings (heterodimerization). This leads to phosphorylation of the intracellular tyrosine kinase residues which serve as docking sites for various effectors and transcription factors that ultimately modulate various biological responses, such as proliferation, survival, migration, and differentiation. Different ligands bind specific receptors based on their binding affinities. This results in a variety of dimerization combinations and, in turn, activation of diverse intracellular pathways. It is noteworthy that *erbB2* has no defined ligand and, due to its conformation design, is the preferred dimerization partner of all other erbB receptors. Furthermore, an *erbB2* heterodimer is characterized by a stronger and more diverse signaling potential than other erbB dimers [21]. *erbB2* overexpression was, indeed, found in various cancers (including breast, ovarian, and endometrial) to be associated with cancer cell proliferation, poor survival, and resistance to therapy [12, 22–25].

## 5. Trastuzumab

It is, therefore, not surprising that *erbB2* has been the target of immunotherapy by monoclonal antibodies (mAb) in a number of cancers. Most notably, 25 to 30 percent of breast cancers overexpress *erbB2*, and have been targeted for mAb therapy. Trastuzumab (Herceptin, Genentech, South San Francisco, Calif) a humanized mAb of the IgG1 family, was approved by the FDA in 1998 for treatment of metastatic breast cancers that overexpress *erbB2* in combination with standard chemotherapy. 

Whereas Trastuzumab is alleged to inhibit downstream signal transduction, ultimately modulating proliferation and apoptosis, its principal mechanism of action is believed to be through recruiting host immune cells (Natural Killer cells), and setting off an antibody-dependent cell-mediated cytotoxicity (ADCC) process [26–28]. This is dependent on receptor overexpression. Based on the breast cancer model, primary USC cell lines in culture challenged with peripheral blood lymphocytes in the presence of Herceptin in a standard 5-hour-chromium-release cytotoxicity assay endured significant killing, up to 75%, compared to control. This was mainly attributed to Herceptin-induced antibodydependent cell-mediated cytotoxicity reaction. Herceptin also had an anti-proliferative effect on those cell cultures [14, 29].

## 6. Trastuzumab in Breast Cancer

In a landmark phase III study, of advanced breast cancer, by Slamon et al. [30], trastuzumab was found to reduce the relative risk of death by 20% at a median followup of 30 months in the group of patients who received chemotherapy and trastuzumab compared to the group who received chemotherapy alone. The addition of trastuzumab to either anthracyclines plus cyclophosphamide or a taxane was also associated with a longer time to disease progression, a high rate of objective response, and a longer duration of response [30]. Eligible patients were *erbB2* 2+ and 3+ overexpressors as determined by immunohistochemistry (IHC). Greater efficacy was noted in IHC 3+ patients compared to the overall population. Subsequently, trastuzumab was consistently shown to significantly improve disease free survival (up to 50%) in multiple other studies when used in combination with multiple other chemotherapies, including taxanes and platinum compounds [31, 32]. The National Surgical Adjuvant Breast and Bowel Project (NSABP) trial B-31 and the North Central Cancer Treatment Group trial N9831 results led to the approval of adjuvant trastuzumab therapy in operable breast cancer. The use of trastuzumab plus chemotherapy in this setting was associated with a 33% reduction in the risk of death (*P* = 0.015) [33]. 

The sentinel indication for Herceptin therapy, namely, *erbB2* positivity, was determined in various trials by either IHC or fluorescent insitu hybridization (FISH). While IHC shows *erbB2* receptor overexpression in cancer specimens, FISH confirms *erbB2* gene amplification. The concordance of those two techniques was retrospectively analyzed and was generally found to be 82% [34]. Concordance between IHC 3+ and FISH-positive tumors was even greater (89%). However, concordance between IHC 2+ tumors and FISH positivity was 24%, begging the question if confirmation of IHC 2+ by FISH would ensure better results.

## 7. Trastuzumab in Endometrial Cancer

A phase II study of single-agent trastuzumab in advanced/recurrent endometrial cancer patients of any histology has recently been reported from the GOG [35]. This study was not able to demonstrate single agent activity of trastuzumab against endometrial carcinoma patients harboring tumors with HER2/neu overexpression. Such results, however, have recently been challenged due to the many shortcomings in the design of the GOG181b study [36]. Moreover, evidence of trastuzumab clinical activity in a handful of heavily pretreated endometrial carcinoma patients has been recently reported as case reports in the medical literature. Consistent with this view, Santin et al. recently reported on two endometrial cancer patients treated with Herceptin. The first case had a stage IIIA platinum refractory G3 endometrioid tumor with IHC 3+ *erbB2* overexpression. The second case had a stage IIIC USC that persisted after surgery and adjuvant pelvic as well as extended field radiation. This patient had an IHC 2+ *erbB2* overexpression. Both cases received salvage treatment with Herceptin with chemotherapy in the former, and as a single agent in the latter. Both cases achieved significant partial response, stable disease, and a substantial sustained decrease in CA125 [37]. In another case report, Jewell et al. reported similar success with Herceptin therapy in combination with chemotherapy in a 72-year-old patient with stage IIIA grade 2 endometrioid adenocarcinoma that recurred after surgery and adjuvant radiation therapy. The tumor showed IHC 3+ *erbB2* overexpression [38]. Finally, Villella et al. reported on two recurrent USC patients with advanced disease and 3+ staining by IHC. When treated with Herceptin, one patient achieved a complete response, and the other had stable disease [39]. Trastuzumab therapy was well tolerated in these patients and, consistent with these results, the single agent use of trastuzumab in a Phase II study of advanced/recurrent endometrial cancer patients by the GOG did not identify any new toxicities or an increased frequency of currently reported toxicities of trastuzumab [35]. In this regard, the major potential toxicities associated with trastuzumab use may include infusion reactions, embryo-fetal toxicity, pulmonary toxicity, and cardiotoxicity. Importantly, the incidence of congestive heart failure and cardiac dysfunction in breast cancer patients have been shown to range from 0.4–3.8% in the major adjuvant trastuzumab trials and to be higher in patients receiving trastuzumab with anthracycline containing chemotherapy regimens [30–33]. 

The above data support the hypothesis that Herceptin may very well be an attractive and viable treatment option for advanced stage USC tumors that overexpress the *erbB2* receptor. This constitutes the merit of a multi-institutional randomized trial due to open soon in the United States (*clinicaltrials.gov/ct2/show/NCT01367002*). The primary objective of this phase II study is to evaluate whether the addition of trastuzumab (Herceptin) to paclitaxel and carboplatin chemotherapy improves progression free survival (PFS) when compared to paclitaxel and carboplatin alone in stages III-IV and recurrent USC patients overexpressing HER2/neu at 3+ level by IHC or positive by FISH. The secondary objectives of the study include (a) assess objective response rate (ORR), (b) assess overall survival (OS), and (c) assess the safety profile of trastuzumab in USPC patients. The exploratory/correlative objectives of the study include (a) determine peripheral blood natural killer (NK) cell numbers and activity in HER2/neu+ USC patients to provide a basis for assessing the possible therapeutic contributions of immune mechanisms of action of trastuzumab, (b) study HER2/neu extracellular domain (ECD) circulating levels in the plasma of USC patients overexpressing HER2/neu before, during and after treatment to elucidate whether changes in HER2/neu ECD would predict response to trastuzumab, and (c) determine whether CA-125 levels correlate with disease activity in advanced and/or recurrent disease. As predicted in 2003, the Achilles heel of USC may soon be exposed [40].
